# Naphthenic acid fraction components from oil sands process‐affected water from the Athabasca Oil Sands Region impair murine osteoblast differentiation and function

**DOI:** 10.1002/jat.4370

**Published:** 2022-08-16

**Authors:** Robert M. Gutgesell, Laiba Jamshed, Richard A. Frank, L. Mark Hewitt, Philippe J. Thomas, Alison C. Holloway

**Affiliations:** ^1^ Department of Obstetrics and Gynecology McMaster University Hamilton ON Canada; ^2^ Water Science and Technology Directorate Environment and Climate Change Canada Burlington ON Canada; ^3^ Environment and Climate Change Canada National Wildlife Research Centre Ottawa ON Canada

**Keywords:** glucocorticoid receptor, naphthenic acids, oil sands process‐affected water, osteoblast, osteoblast differentiation, osteoblastogenesis

## Abstract

The extraction of bitumen from surface mining in the Athabasca Oil Sands Region (AOSR) produces large quantities of oil sands process‐affected water (OSPW) that needs to be stored in settling basins near extraction sites. Chemical constituents of OSPW are known to impair bone health in some organisms, which can lead to increased fracture risk and lower reproductive fitness. Naphthenic acid fraction components (NAFCs) are thought to be among the most toxic class of compounds in OSPW; however, the effect of NAFCs on osteoblast development is largely unknown. In this study, we demonstrate that NAFCs from OSPW inhibit osteoblast differentiation and deposition of extracellular matrix, which is required for bone formation. Extracellular matrix deposition was inhibited in osteoblasts exposed to 12.5–125 mg/L of NAFC for 21 days. We also show that components within NAFCs inhibit the expression of gene markers of osteoblast differentiation and function, namely, alkaline phosphatase (*Alp*), osteocalcin, and collagen type 1 alpha 1 (*Col1a1*). These effects were partially mediated by the induction of glucocorticoid receptor (GR) activity; NAFC induces the expression of the GR activity marker genes *Sgk1* (12.5 mg/L) and *p85a* (125 mg/L) and inhibits GR protein (125 mg/L) and *Opg* RNA (12.5 mg/L) expression. This study provides evidence that NAFC concentrations of 12.5 mg/L and above can directly act on osteoblasts to inhibit bone formation and suggests that NAFCs contain components that can act as GR agonists, which may have further endocrine disrupting effects on exposed wildlife.

## Abstract 2

Surface mining in the Athabasca Oil Sands Region (AOSR) produces large quantities of oil sands process‐affected water (OSPW), constituents of which are known to impair bone health in some organisms. Naphthenic acid fraction components (NAFCs) are thought to be responsible for most of the toxic effect of OSPW, but their effects on bone are unknown. In this study, we demonstrate that NAFCs extracted from the AOSR inhibit osteoblast differentiation and may act via the glucocorticoid receptor.

## 1 INTRODUCTION

The Athabasca Oil Sands Region (AOSR) is one of the world's largest deposits of extractable bitumen (Alberta Energy Regulator, 2015). The process of bitumen extraction uses and recycles water, which becomes oil sands process‐affected water (OSPW); this OSPW is then stored in tailings ponds and cannot be discharged into receiving waters after use (Wu et al., 2019). There is concern that chemicals from bitumen extraction may be affecting development and reproductive health of animals in areas impacted by mining activity; many of these concerns are centered around the toxicity of OSPW (Li et al., 2017). There is further concern that components of OSPW are able to migrate beyond tailings ponds containment structures into nearby waterbodies and wetlands (Hewitt et al., 2020).

OSPW contains a variety of compounds including naphthenic acid fraction components (NAFCs) which are a large and diverse group of organic acids that are natural components of bitumen (Clemente & Fedorak, 2005). NAFCs are generally considered to be the most toxic class of organic compound in OSPW (Li et al., 2017). As a result, the toxicity of NAFCs has been widely studied. Indeed, there is evidence demonstrating the toxicity of OSPW on invertebrates (Bartlett et al., 2017) and fish (Mahaffey & Dubé, 2016; Marentette et al., 2015, 2017). Although few studies have investigated the effects that chronic exposures to NAFCs have on major organ systems in mammals (Clemente & Fedorak, 2005; Li et al., 2017), there is evidence that OSPW and NAFCs can act as endocrine disrupting compounds. Endocrine effects of NAFCs that have been observed include alterations in sex hormone and glucocorticoid production (Knag et al., 2013; Lister et al., 2008), impaired development and reproductive success, and reduced secondary sexual characteristics in animals exposed to NAFCs (Kavanagh et al., 2013; Li et al., 2019; Philibert et al., 2019; Rogers et al., 2002).

More recently, in a study investigating the baculum strength of animals in the AOSR, lower baculum strength was observed in river otters (*Lontra canadensis*) collected in areas with high levels of mining activity compared with those collected in areas far from mining activity (Thomas et al., 2021). A decrease in baculum bone material properties can reduce the reproductive fitness of male mammals, thereby increasing pressures on local river otter populations (Brassey et al., 2018; Sonne et al., 2015; Stockley et al., 2013). Similar to Thomas et al. (2021), previous studies in other free‐ranging mammals have also shown that exposure to endocrine disrupting chemicals can lower baculum bone mineral density (BMD) (Sonne et al., 2015). Although there is limited information available regarding the effects of OSPW or NAFCs on mammalian bone, a study in fathead minnow larvae reported that both a commercial technical naphthenic acid mixture and NAFCs of tailings water from the AOSR induced skeletal deformities and altered gene networks related to chondrocyte development and endochondral ossification (Marentette et al., 2015). These data suggest that it is biologically plausible that NAFCs may also impact mammalian bone development.

Bone strength is maintained throughout life by the process of remodeling, which allows bone to adapt and repair microdamage through the resorption of old bone and deposition of new bone matrix (Seeman, 2003). This process is regulated by endocrine factors and paracrine signaling between osteocytes, osteoblasts, and osteoclasts (Hauge et al., 2001). Osteoblasts synthesize new bone matrix on bone‐forming surfaces as they differentiate from osteoblast progenitor cells during the formation phase of the remodeling cycle (Komori, 2016). The primary endocrine pathways regulating BMD are parathyroid hormone (PTH) (Silva & Bilezikian, 2015; Stein et al., 2013), insulin‐like growth factor 1 (IGF‐1) (McClung et al., 2014; Plotkin & Bellido, 2016), and glucocorticoids (Rauch et al., 2010; Weinstein et al., 1998). Glucocorticoid receptor (GR) activation in osteoblasts inhibits differentiation and production of extracellular matrix (ECM) (Rauch et al., 2010). Further, the use of synthetic glucocorticoids like dexamethasone is also the most common cause of secondary osteoporosis (Briot & Roux, 2015). Exposure to OSPW has been found to increase plasma cortisol levels in fish (Lister et al., 2008), but studies have yet to investigate whether chemicals in OSPW act as agonists for PTH, IGF‐1, or glucocorticoid receptors. The goals of this study were (1) to investigate the effects of the NAFC extracted from OSPW from an active mining operation within the AOSR (Frank et al., 2006) on the osteogenic potential of mouse pre‐osteoblastic (MC3T3‐E1) cells and (2) to determine if the underlying mechanism is glucocorticoid receptor dependent.

## 2 MATERIALS AND METHODS

### 2.1 NAFC preparation

Naphthenic acid extracts were isolated from tailings pond water collected from Syncrude Canada Ltd. West In‐pit settling basin in Fort McMurray, Alberta, Canada, in 2009, using a procedure described in (Frank et al., 2006). An NAFC stock solution was prepared and stored in glass vessels in darkness and preserved in 0.05 M NaOH with a final concentration of 2504 mg/L determined via liquid chromatography/quadrupole mass spectrometry with time‐of‐flight detection (LC/QTofF) as previously described in Bartlett et al. (2017) and Marentette et al. (2015, 2017).

### 2.2 MC 3T3‐E1 cell culture and treatment

MC 3T3‐E1 subclone 4 mouse newborn fibroblasts (ATCC, Manassas, VA, USA) were maintained in ascorbic acid‐free alpha minimum essential medium (aMEM) with ribonucleosides, deoxyribonucleosides, 2 mM L‐glutamine, and 1 mM sodium pyruvate (Gibco, Waltham, MA, USA), supplemented with 10% fetal bovine serum (Hyclone, Logan, UT, USA) and 1% P/S. When cells were 70% confluent, they were seeded into six‐well plates (2000 cells/cm^2^) and left to grow until they were 100% confluent. Forty‐eight hours after reaching full confluence (i.e., 100%; day 0), differentiation was induced with 100 μM L‐ascorbic acid (EMD, Mississauga, ON, Canada) and 2 mM β‐glycerophosphate (Sigma, St. Louis, MO, USA) (Wang et al., 1999) added to the supplemented αMEM media (differentiation media). The MC 3T3‐E1 subclone 4 cell line used in this study is a commonly used model to study osteoblastogenesis and reliably generates a mineralized extracellular matrix after 21 days of treatment in an appropriate osteogenic medium (Hwang & Horton, 2019); days 7 and 14 represent early and middle stages of differentiation, respectively.

To investigate whether NAFCs inhibits osteoblastogenesis, cells were treated beginning on day 0 with 125 mg/L, 25 mg/L, 12.5 mg/L, and 1.25 mg/L of the NAFC mixture, which was diluted in differentiation media (described above). These concentrations encompass the range of NAFCs, which have been reported in surface waters, wetlands, and tailings ponds in the AOSR (Vander Meulen et al., 2021). Dexamethasone (1 μM, Sigma), a glucocorticoid receptor agonist, was used as a positive control to assess the effects of GR activation during differentiation (Rauch et al., 2010). The pH of the media was adjusted to be similar across all groups using NaOH or HCl after the addition of NAFCs. Cells were treated for 7, 14, or 21 days after the initiation of differentiation; media was changed every 4 days.

### 2.3 Cell viability

Cells were seeded and treated in 96‐well plates following the protocols described above. After 7 and 14 days of treatment, 10% alamarBlue reagent (BioRad Laboratories, Hercules, CA, USA) was added to fresh media and cells were incubated for 2 h. Absorbance was then read at 570 nm in a plate reader (Synergy H1 microplate reader, Norgen BioTek, Thorold, ON, Canada) with a reference read at 600 nm. Treatments were considered to be cytotoxic if the average absorbance for the group was below 80% of the control group.

### 2.4 Real‐time quantitative PCR

After 7, 14, or 21 days of treatment, representing early, middle, and final stages of differentiation, respectively, cells were washed with phosphate‐buffered saline (PBS), and RNA was extracted using TRIzol reagent (Invitrogen, Waltham, MA, USA). RNA concentrations were determined using the NanoDrop One Microvolume UV‐Vis Spectrophotometer (Thermo Fisher Scientific). Complementary DNA (cDNA) was made from 2 μg of mRNA using the High‐Capacity cDNA Reverse Transcription Kit (Applied Biosystems, Waltham MA, USA) as per the manufacturer's instructions. Real‐time quantitative PCR (RT‐qPCR) was carried out using PerfeCTa SYBR Green FastMix (Quanta Biosciences, Gaithersburg, MD, USA) on the CFX384 Touch Real Time PCR Detection System (Bio‐Rad Laboratories). The PCR cycling settings included polymerase activation (95°C for 10 m), followed by 40 cycles of denaturing (95°C for 15 s) and annealing/elongation (60°C for 1 m). Levels of gene expression were generated using the 2(−ΔΔC(T)) method (Livak & Schmittgen, 2001) and normalized using the geometric means of two reference genes: tyrosine 3‐monooxygenase/tryptophan 5‐monooxygnase activation protein zeta (*Ywhaz*) and peptidylprolyl isomerase A (*Ppia*). Gene targets for osteoblast differentiation: alkaline phosphatase (*Alp*), collagen type 1 alpha 1 chain (*Col1a1*), *Osteocalcin*, and runt‐related transcription factor 2 (*Runx2*), glucocorticoid receptor activity: serum/glucocorticoid‐inducible kinase 1 (*Sgk1*), receptor activator of nuclear factor kappa‐B ligand (*Rankl*), osteoprotegerin (*Opg*), and phosphatidylinositol 3‐kinase regulatory subunit alpha (*p85a*) were measured. Primer sequences are presented in Table 1.

**TABLE 1:** Table of primers

Gene	Forward/reverse	Sequence	Accession no.
ALP	Forward	5′‐AGCAGGTTTCTCTCTTGGGC‐3′	NM_007431.3
ALP	Reverse	5′‐GGTGCTTTGGGAATCTGTGC‐3′	
COL1A1	Forward	5′‐GGTGGGGTGGGAAGGAATTT‐3′	NM_007742.4
COL1A1	Reverse	5′‐GGTCTAGGGAGCATCTCAGC‐3′	
IGF‐1	Forward	5′‐CAGGAGGGTGCAACATCAGA‐3′	NM_010512.5
IGF‐1	Reverse	5′‐GTGGCATCCCAGTGACAGAT‐3′	
LGR5	Forward	5′‐CAAGCCCTGTGAGCACCTAT‐3′	NM_010195.2
LGR5	Reverse	5′‐TCTGAACACGGTCAAAGCCA‐3′	
OPG	Forward	5′‐GGAACCCCAGAGCGAAACAC‐3′	NM_008764.3
OPG	Reverse	5′‐GCCAAATGTGCTGCAGTTCG‐3′	
Osteocalcin	Forward	5′‐TTCTGCTCACTCTGCTGACC‐3′	NM_007541.3
Osteocalcin	Reverse	5′‐TATTGCCCTCCTGCTTGGAC‐3′	
p85a	Forward	5′‐CAAAGCGGAGAACCTATTGC‐3′	NM_001024955.2
p85a	Reverse	5′‐ATAGCAGCCCTGCTTACTGC‐3′	
PPIA	Forward	5′‐GACAAAGTTCCAAAGACAGCAGAA‐3′	NM_008907.2
PPIA	Reverse	5′‐CCAAATCCTTTCTCTCCAGTGC‐3′	
RANKL	Forward	5′‐AGGGAGCACGAAAAACTGGT‐3′	NM_011613.3
RANKL	Reverse	5′‐GGAAGGGTTGGACACCTGAA‐3′	
RUNX2	Forward	5′‐GCCTCCAGCACCCTATACCC‐3′	NM_001271631.1
RUNX2	Reverse	5′‐CACATAGGTCCCCATCTGCC‐3′	
SGK1	Forward	5′‐GGGTGCCAAGGATGACTTTA‐3′	NM_001161845.2
SGK1	Reverse	5′‐CTCGGTAAACTCGGGATCAA‐3′	
YwHAZ	Forward	5′‐GGGGTGATTGGCAAAAGGTA‐3′	NM_001356569.1
YwHAZ	Reverse	5′‐CGACTTGGAAGCACAGAACT‐3′	

### 2.5 ALP staining and enzyme activity assay

Alkaline phosphatase (ALP) is major marker of the early and middle stage of osteoblast differentiation (Lee et al., 2017). To visualize ALP abundance, after 14 days of treatment, cells were washed with PBS and fixed in 10% formalin for 10 min. The fixed cells were incubated in a solution containing 1 mg/ml Fast Blue RR salt and 0.4 mg/ml naphthol AS‐MX phosphate disodium salt (Thermo Fisher Scientific, Waltham, MA, USA) at pH 8.4 for 15 min. The reactions were stopped by rinsing with deionized water, and cells were photographed under a light microscope (Motic Microscopes, Xiamen, China). To measure ALP activity, an enzymatic ALP activity assay (Abcam, Cambridge, UK) was used according to the instructions provided by the manufacturer. Briefly, cells were harvested with a cell lysis solution, a p‐nitrophenyl phosphate (pNPP) phosphatase substrate was applied to the samples, and absorbance was read at 405 nm using a microplate reader (Norgen BioTek). Absorbance data were corrected to background and compared with control.

### 2.6 Alizarin Red S staining of MC3T3‐E1 osteoblasts

To measure calcium deposition, after 21 days of treatment, cells were washed with Dulbecco's phosphate‐buffered saline (DPBS), then fixed with 10% formalin and stained with 2% Alizarin Red S (ARS) solution (EMD) for 15 min. The ARS solution was removed, and the cells were then washed five times with water. Representative images were taken using phase‐contrast microscopy (Motic Microscopes). The dye was extracted using 10% acetic acid, cells were separated, and supernatant was transferred to a 96‐well plate and absorbance was read at 405 nm in a microplate reader (Norgen BioTek). Absorbance data were corrected for background and compared with control.

### 2.7 Immunoblotting

After 21 days of treatment, cells were isolated in lysis buffer (50 mM HEPES, 150 mM NaCl, 100 mM NaF, 10 mM sodium pyrophosphate, 5 mM EDTA, 250 mM sucrose, 1 mM dithiothreitol, and 1 mM sodium orthovanadate, with 1% Triton X and one tablet of cOmplete Protease Inhibitor Cocktail [Roche, Basil, Switzerland] per 50 ml) then stored at −80°C until analysis. Protein concentration was determined with the Pierce BCA Protein Assay kit (Thermo Fisher Scientific). Lysates were then diluted with Laemmli buffer and run on a 10% polyacrylamide gel (Bio‐Rad Laboratories) to separate proteins based on size. Samples were then transferred to a polyvinylidene difluoride (PVDF) membrane and blocked in 5% BSA for 1 h at room temperature (20–25°C). Membranes were incubated with primary antibody (1:1000) overnight at 4°C. Appropriate secondary antibodies were used at a concentration of 1:10,000. Bound antibodies were detected using Clarity Western ECL Substrate (BioRad Laboratories). For primary antibody information, see Table 2.

**TABLE 2:** Table of antibodies

Antibody	Manufacturer	Product number
β‐actin	Cell Signaling Technology	4967
GR	Santa Cruz Biotechnology	sc‐1004
Osteocalcin	abcam	ab93876
RANKL	R&D Systems	AF462
RUNX2	abcam	ab76956

### 2.8 Statistical analysis

All statistical analyses were carried out using GraphPad Prism 9.2 (GraphPad Software Inc, San Diego, CA, USA). One‐way analysis of variance (ANOVA) was performed to detect significant interactions between experimental groups and negative control groups. Multiple comparisons were analyzed following Dunnett's multiple comparisons post hoc tests. Positive control groups (dexamethasone) were compared with negative control groups using a Student's *t* test. Any *p* value lower than 0.05 was considered significant. All results are presented as means ± SEM.

## 3 RESULTS

### 3.1 NAFCs inhibits osteoblastogenesis in murine pre‐osteoblasts

**FIGURE 1:**
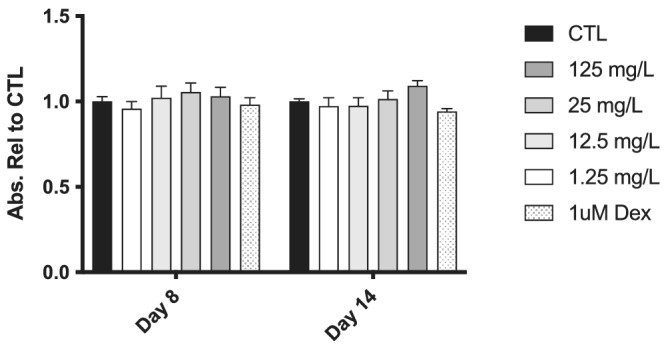
Effects of NAFCs on cell viability. Cell viability of MC3T3‐E1 osteoblastic cells after 8 and 14 days of treatment with of concentrations of NAFC from 1.25 mg/L to 125 mg/L and 1 μM dexamethasone positive control. Each value is the mean ± SEM (*n* = 6). *p < 0.05, compared with the corresponding control group at each time point.

**FIGURE 2:**
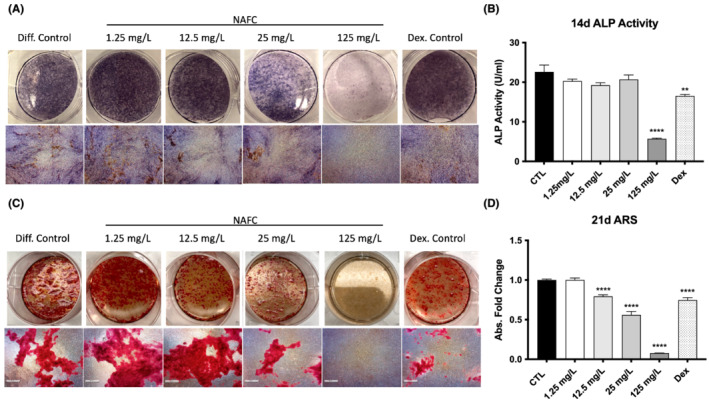
NAFCs inhibits osteoblast differentiation in MC3T3‐E1 cells in a dose‐dependent manner. (A) ALP staining representative images in MC 3T3‐E1 cells treated for 14 days with 1.25–125 mg/L NAFCs and 1 μM dexamethasone positive control. (B) ALP activity of MC 3T3‐E1 cells treated for 14 days with 1.25–125 mg/L NAFC and 1 μM dexamethasone positive control. (C–D) ARS calcium staining assay results and representative images in MC 3T3‐E1 cells treated for 21 days with 1.25–125 mg/L NAFC and 1 μM dexamethasone positive control. Each value is the mean ± SEM (*n* = 6). *p < 0.05, **p < 0.01, ***p < 0.001, ****p < 0.0001, treated groups compared with the differentiation control group.

Treatment with all doses of NAFCs (1.25 mg/L to 125 mg/L) for 8 and 14 days had no impact on the viability of MC3T3‐E1 pre‐osteoblast cells (Figure 1). Following 14 days of treatment with NAFC, ALP activity was assessed by ALP activity assay and staining. Treatment with 125 mg/L NAFCs as well as 1 μM dexamethasone led to significantly lower ALP activity than in control cells (Figure 2A,B). The deposition of calcium in a mineralized ECM is a common functional marker for the late stages of osteogenic differentiation. We measured the amount of calcium in 3T3‐E1 cells following 21 days of treatment with NAFCs using Alizarin Red S (ARS) calcium staining. Treatment with 12.5–125 mg/L of NAFCs resulted in a dose‐dependent reduction in extracellular calcium deposition (Figure 2C,D).

**FIGURE 3:**
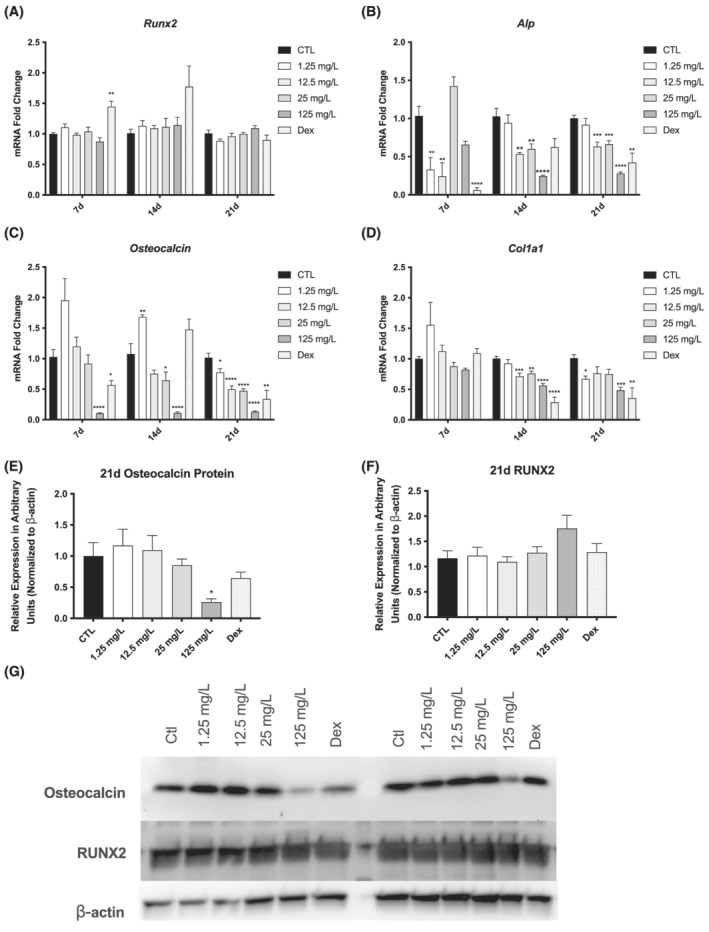
Effects of NAFC on osteoblast markers during differentiation of MC3T3‐E1 cells. (A–D) Expression of osteoblastic marker genes *Runx2*, *Alp*, *osteocalcin*, and *Col1a1* in MC 3T3‐E1 cells treated for 7, 14, and 21 days with 1.25–125 mg/L NAFC and 1 μM dexamethasone positive control. (E–G) Protein concentration and western blot representative images of osteocalcin and RUNX2 in MC 3T3‐E1 cells treated for 21 days with 1.25–125 mg/L NAFC and 1 μM dexamethasone positive control. The order of the lanes is consistent, though the alignment of the blots may vary. Each value is the mean ± SEM (*n* = 6). *p < 0.05, **p < 0.01, ***p < 0.001, ****p < 0.0001, treated groups compared with the differentiation control group.

To further evaluate the effect that NAFCs has on osteoblastogenesis, the expression of markers of osteoblast differentiation *Runx2*, *Alp*, *osteocalcin*, and *Col1a1* were measured after 7, 14, and 21 days of exposure. NAFCs had no impact on *Runx2* gene or protein expression at any time point (Figure 3A,F), but the 12.5–125 mg/L doses of NAFCs induced significant reductions in the expression of *Alp*, *osteocalcin*, and *Col1a1* genes at the 14‐ and 21‐day time points relative to control treated cells (Figure 3B–D). The lowest dose of NAFCs significantly inhibited *Alp* gene expression at 7 days only. At 14 and 21 days, NAFCs exposures resulted in significantly lower Alp expression at 12.5, 25, and 125 mg/L doses (Figure 3B). Similarly, 125 mg/L of NAFCs significantly inhibited *osteocalcin* expression at all time points, and by 21 days, all doses of NAFCs had significantly lower *osteocalcin* expression (Figure 3C). Accordingly, we observed significantly lower osteocalcin protein expression in cells exposed to 125 mg/L of NAFCs than control cells (Figure 3E). *Col1a1* expression was unchanged after 7 days of NAFCs treatment, but after 14 days, cells treated with ≥12.5 mg/L of NAFCs exhibited significantly lower *Col1a1* expression than control cells, which then persisted to 21 days in cells treated with 125 mg/L of NAFCs (Figure 3D).

### 3.2 NAFCs inhibition of osteoblastogenesis is mediated by glucocorticoid receptor activity

**FIGURE 4:**
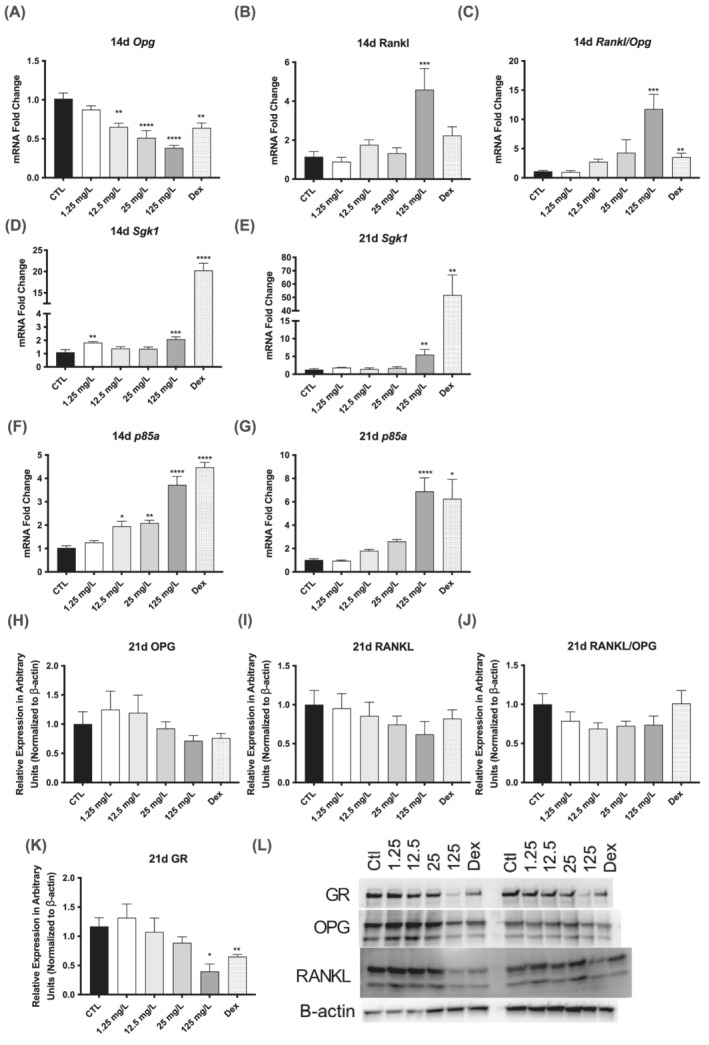
NAFCs induce glucocorticoid receptor activity in MC3T3‐E1 cells. Expression of glucocorticoid receptor activity markers in MC3T3‐E1 cells treated with 1.25–125 mg/L NAFCs for 14 and 21 days with 1 μM dexamethasone positive control. (A–C) RNA levels of *Opg* and *Rankl* and *Rankl/Opg* ratio after 14 days of NAFCs treatment. (D–G) RNA levels of *Sgk1* and *p85a* after 14 and 21 days of NAFCs treatment. (H–J) Protein levels of OPG, RANKL, and RANKL/OPG ratio following 21 days of NAFCs treatment. (K) Protein levels of GR protein following 21 days of NAFCs treatment. (L) Western blotting representative images. Each value is the mean ± SEM (*n* = 6). *p < 0.05, **p < 0.01, ***p < 0.001, ****p < 0.0001, treated groups compared with the differentiation control group.

Given that GR activity has a common and strong inhibitory effect on osteoblastogenesis, we sought to determine if GR was implicated in the mechanism by which NAFCs inhibits osteoblastogenesis. We measured the gene expression of downstream markers of GR activity *Opg*, *Rankl* after 14 days of NAFCs treatment and *Sgk1* and *p85a* after 14 and 21 days of treatment. We observed a dose‐dependent decrease in *Opg* expression as well as a significant increase in *Rankl* expression in the 125 mg/L NAFCs treated cells, resulting in a significant increase in the *Rankl*/*Opg* ratio at 14 days (Figure 4A–C). We also observed significant increases in *Sgk1* gene expression after both 14 and 21 days of treatment with 125 mg/L of NAFCs and after 14 days of treatment with 1.25 mg/L of NAFC (Figure 4D,E). *P85a* expression was significantly higher with 14 days of NAFCs treatment in a dose‐dependent manner, an effect which persisted at 21 days (Figure 4F,G). We then measured protein markers of GR activity after 21 days of treatment with NAFCs and found that there was no change in OPG or RANKL protein expression with NAFCs or the 1 μM dexamethasone GR agonist positive control, but there was a significant decrease in GR protein expression in cells treated with both 125 mg/L of NAFCs and dexamethasone (Figure 4H–L). These results are consistent with an induction of GR activity by NAFCs functioning as GR agonists.

## 4 DISCUSSION

Although there are numerous studies which have investigated the toxicological effects of environmental contaminants derived from oil and gas production (Li et al., 2017; Rodríguez‐Estival & Smits, 2016), few of these have focused on bone health. Three studies have found skeletal deformities caused by OSPW or light oil fraction on bone in fish (Danion et al., 2011; Loughery et al., 2019; Marentette et al., 2015), and another found that river otters living closer to AOSR mining projects had generally poorer baculum bone material properties than otters farther from mining activity (Thomas et al., 2021). In the present study, we found that treatment with NAFCs derived from the AOSR are sufficient to inhibit osteoblast differentiation, determined by measuring calcified ECM, ALP activity, and gene and protein markers of osteoblastogenesis. We also found that NAFCs exposure at concentrations as low as 12.5 mg/L alters the expression of components of GR signaling similar to what is seen with a known GR agonist dexamethasone.

In the current study, we found that treatment with NAFCs inhibited the differentiation of osteoblast precursor cells into mature osteoblasts. Treatment with 12.5–125 mg/L of NAFCs for 21 days significantly decreased the deposition of extracellular matrix in differentiating osteoblasts (Figure 2D), indicating an inhibition in the differentiation and primary function of these cells. ALP enzyme activity, a major marker of osteogenic differentiation (Lee et al., 2017), was reduced with 125 mg/L of NAFCs treatment for 14 days (Figure 2B), and we observed corresponding reductions in Alp gene expression at doses of 12.5 mg/L and higher at 14 and 21 days (Figure 3B). We also observed lower expression of *osteocalcin*, a marker of osteoblast differentiation that is specifically expressed by mature osteoblasts (Huang et al., 2007) at most doses of NAFCs treatment at 14 and 21 days (Figure 3C), as well as lower osteocalcin protein expression after 21 days of treatment with 125 mg/L of NAFCs (Figure 3E), further corroborating an inhibition in osteoblastogenesis. *Col1a1* is another gene marker of osteoblast differentiation that is expressed throughout differentiation beginning at the mature osteoprogenitor phase (Huang et al., 2007), and we see reduced Col1a1 expression with most doses of NAFCs at 14 and 21 days of treatment (Figure 3D). These results are consistent with an inhibition of osteoblastogenesis by NAFCs that is likely occurring later in the differentiation process. Because we saw decreased mineralization, a marker of osteoblast differentiation and decreased osteocalcin expression (a marker of mature osteoblasts) at 21 days following NAFCs exposure, we expected a decrease in the expression of Runx2, which is one of the most important osteogenic transcription factors and an important marker of early osteoblastogenesis (Liu & Lee, 2013) with NAFCs treatment. However, our observation that there was no change in Runx2 expression (Figure 3A,F) suggests that NAFCs are inhibiting osteoblastogenesis downstream of the regulation of Runx2 expression. These findings are in line with previous work in which exposure to the water‐soluble fraction of light cycle oil, which contains several petroleum related compounds, resulted in lower bone mineralization in sea bass (Danion et al., 2011). Further, research from our group and others has demonstrated that commercial NA mixtures and NA fraction components of OSPW increase spinal deformities and skeletal gene networks of chondrocyte development and endochondral ossification, osteoclast activation and function in fathead minnow larvae (Loughery et al., 2019; Marentette et al., 2015). Similarly, the data presented here demonstrate that NAFCs have a direct effect on osteoblasts.

In this study, we also show that NAFCs can activate GR in a manner similar to dexamethasone. Glucocorticoids like dexamethasone inhibit osteoblast mineral deposition and ALP activity through inducing GR activity (Rauch et al., 2010). NAFCs and dexamethasone treatment resulted in similar changes in the expression of GR protein (Figure 4K) suggesting NAFCs is also acting as a GR agonist (Kuo et al., 2012; Xu et al., 2019). We also observed increases in the expression of gene markers of GR activity *p85a* and *Sgk1* after 21 days of treatment with NAFCs treatment (Figure 4D–G). Sgk1 plays an important role in calcification of vascular muscle cells (Poetsch et al., 2020) and is required for the late stages of osteoblastogenesis (Kim et al., 2022). Other markers, RANKL and OPG are osteoblast‐derived proteins that are important for regulating bone mass and are disrupted with GR activity (Humphrey et al., 2006). We observed a decrease in *Opg* expression and an increase in *Rankl* expression and increased ratio of *Rankl*/*Opg* with NAFCs treatment (Figure 4A–C), which is further evidence that NAFCs is likely inducing GR activity in the middle stages of differentiation. However, these changes in gene expression did not translate to significantly different RANKL and OPG protein expression after 21 days of NAFCs exposure (Figure 4H–J). Taken together, these findings suggest that NAFCs inhibits osteoblastogenesis by acting on the GR although this remains to be confirmed by using pharmacological inhibition or knockdown of GR signaling pathways.

Previous studies that have investigated the effects of NAFCs on gene markers of skeletal development did not measure the expression of the markers of osteoblastogenesis: *Alp*, *Col1a1*, or *osteocalcin* (Loughery et al., 2019). The lower expression of *Alp*, *Col1a1*, and *osteocalcin* that we observed (Figure 3B–D) is consistent with what others have reported in osteoblasts or mesenchymal stem cells treated with glucocorticoids (Chen et al., 2018; Li et al., 2015). Due to the fact that the NAFCs is a mixture of several thousand organic acids, various components of the NAFCs mixture could be functioning through multiple pathways that contribute to the totality of responses observed with the whole mixture. It is likely that some components of the NAFCs may be inhibiting osteoblast differentiation through other mechanisms, in addition to those acting via the GR pathway. NAFCs from other sources have previously been shown to alter other pathways that can affect BMD, including altered sex hormone function (Knag et al., 2013; Lister et al., 2008) and increased inflammatory cytokine expression (Garcia‐Garcia et al., 2011); the effects of our NAFC mixture on these pathways remains to be determined. Our data suggest that NAFC is acting through GR. However, NAFCs are complex mixtures that could also act through other pathways to inhibit osteoblastogenesis such as PTH, wingless‐related integration site (Wnt), or peroxisome proliferator‐activated receptor gamma (PPARγ) among others (Bateman et al., 2017; Silva & Bilezikian, 2015).

Although our findings are consistent with the hypothesis that NAFCs exposure has the potential to inhibit bone formation, this study was conducted in osteoblasts, which only represent one bone cell lineage. Moreover, using this model, we cannot assess if the effects of NAFCs on bone are reversible. Another major limitation of studies investigating the toxicological effects of NAFCs is the challenge of measuring the tissue concentrations and compositions of NAFCs in wildlife (Bartlett et al., 2017; Marentette et al., 2015, 2017). Although our findings utilized concentrations of NAFCs that have been reported in surface water, industrially affected wetlands, and tailings ponds in the AOSR (Vander Meulen et al., 2021), the concentration at the tissue level is uncertain. However, the concentrations of NAFCs used in this study are similar to those used in other toxicological studies. Despite the limitations of this study, our finding that osteoblast differentiation is inhibited with exposure to NAFCs may have important implications for the bone strength of exposed animals and warrants the study of the effects of NAFCs exposures in vivo.

## CONFLICT OF INTEREST

The authors have no conflict of interest to report.

## DISCLAIMER

The views in this paper are only held by the authors and are not representative of the official policy of the authors' individual affiliations.

## Data Availability

Research data are not shared.

## References

[jat4370-bib-0001] Alberta Energy Regulator . 2015. Alberta's energy reserves 2014 and supply/demand outlook 2015–2024: Calgary. Alberta, Canada, Alberta Energy Regulator Statistical Series ST98‐2015.

[jat4370-bib-0002] Bartlett, A. J. , Frank, R. A. , Gillis, P. L. , Parrott, J. L. , Marentette, J. R. , Brown, L. R. , Hooey, T. , Vanderveen, R. , McInnis, R. , Brunswick, P. , Shang, D. , Headley, J. V. , Peru, K. M. , & Hewitt, L. M. (2017). Toxicity of naphthenic acids to invertebrates: Extracts from oil sands process‐affected water versus commercial mixtures. Environmental Pollution (Barking, Essex: 1987), 227, 271–279. 10.1016/j.envpol.2017.04.056 28477551

[jat4370-bib-0003] Bateman, M. E. , Strong, A. L. , McLachlan, J. A. , Burow, M. E. , & Bunnell, B. A. (2017). The effects of endocrine disruptors on adipogenesis and osteogenesis in mesenchymal stem cells: A review. Frontiers in Endocrinology, 7, 171. 10.3389/fendo.2016.00171 28119665 PMC5220052

[jat4370-bib-0004] Brassey, C. A. , Gardiner, J. D. , & Kitchener, A. C. (2018). Testing hypotheses for the function of the carnivoran baculum using finite‐element analysis. Proceedings of the Royal Society B: Biological Sciences, 285, 20181473. 10.1098/rspb.2018.1473 PMC6170803 30232157

[jat4370-bib-0005] Briot, K. , & Roux, C. (2015). Glucocorticoid‐induced osteoporosis. RMD Open, 1(1), e000014. 10.1136/rmdopen-2014-000014 26509049 PMC4613168

[jat4370-bib-0006] Chen, S.‐M. , Peng, Y.‐J. , Wang, C.‐C. , Sui‐Lung, S. , Salter, D. M. , & Lee, H.‐S. (2018). Dexamethasone down‐regulates osteocalcin in bone cells through leptin pathway. International Journal of Medical Sciences, 15(5), 507–516. 10.7150/ijms.21881 29559840 PMC5859774

[jat4370-bib-0007] Clemente, J. S. , & Fedorak, P. M. (2005). A review of the occurrence, analyses, toxicity, and biodegradation of naphthenic acids. Chemosphere, 60(5), 585–600. 10.1016/j.chemosphere.2005.02.065 15963797

[jat4370-bib-0008] Danion, M. , Deschamps, M.‐H. , Thomas‐Guyon, H. , Bado‐Nilles, A. , Le Floch, S. , Quentel, C. , & Sire, J.‐Y. (2011). Effect of an experimental oil spill on vertebral bone tissue quality in European sea bass (*Dicentrarchus labrax* L.). Ecotoxicology and Environmental Safety, 74(7), 1888–1895. 10.1016/j.ecoenv.2011.07.027 21831432

[jat4370-bib-0009] Frank, R. A. , Richard Kavanagh, B. , Burnison, K. , Headley, J. V. , Peru, K. M. , Van Der Kraak, G. , & Solomon, K. R. (2006). Diethylaminoethyl‐cellulose clean‐up of a large volume naphthenic acid extract. Chemosphere, 64(8), 1346–1352. 10.1016/j.chemosphere.2005.12.035 16469358

[jat4370-bib-0010] Garcia‐Garcia, E. , Ge, J. Q. , Oladiran, A. , Montgomery, B. , El‐Din, M. G. , Perez‐Estrada, L. C. , Stafford, J. L. , Martin, J. W. , & Belosevic, M. (2011). Ozone treatment ameliorates oil sands process water toxicity to the mammalian immune system. Water Research, 45(18), 5849–5857. 10.1016/j.watres.2011.08.032 21940034

[jat4370-bib-0011] Hauge, E. M. , Qvesel, D. , Eriksen, E. F. , Mosekilde, L. , & Melsen, F. (2001). Cancellous bone remodeling occurs in specialized compartments lined by cells expressing osteoblastic markers. Journal of Bone and Mineral Research: The Official Journal of the American Society for Bone and Mineral Research, 16(9), 1575–1582. 10.1359/jbmr.2001.16.9.1575 11547826

[jat4370-bib-0012] Hewitt, L. M. , Roy, J. W. , Rowland, S. J. , Bickerton, G. , DeSilva, A. , Headley, J. V. , Milestone, C. B. , Scarlett, A. G. , Brown, S. , Spencer, C. , West, C. E. , Peru, K. M. , Grapentine, L. , Ahad, J. M. E. , Pakdel, H. , & Frank, R. A. (2020). Advances in distinguishing groundwater influenced by oil sands process‐affected water (OSPW) from natural bitumen‐influenced groundwaters. Environmental Science & Technology, 54(3), 1522–1532. 10.1021/acs.est.9b05040 31906621 PMC7003248

[jat4370-bib-0013] Huang, W. , Yang, S. , Shao, J. , & Li, Y.‐P. (2007). Signaling and transcriptional regulation in osteoblast commitment and differentiation. Frontiers in Bioscience: A Journal and Virtual Library, 12, 3068–3092. 10.2741/2296 17485283 PMC3571113

[jat4370-bib-0014] Humphrey, E. L. , Williams, J. H. H. , Davie, M. W. J. , & Marshall, M. J. (2006). Effects of dissociated glucocorticoids on OPG and RANKL in osteoblastic cells. Bone, 38(5), 652–661. 10.1016/j.bone.2005.10.004 16298558

[jat4370-bib-0015] Hwang, P. W. , & Horton, J. A. (2019). Variable osteogenic performance of MC3T3‐E1 subclones impacts their utility as models of osteoblast biology. Scientific Reports, 9, 8299. 10.1038/s41598-019-44575-8 31165768 PMC6549152

[jat4370-bib-0016] Kavanagh, R. J. , Frank, R. A. , Solomon, K. R. , & Van Der Kraak, G. (2013). Reproductive and health assessment of fathead minnows (*Pimephales promelas*) inhabiting a pond containing oil sands process‐affected water. Aquatic Toxicology (Amsterdam, Netherlands), 130–131, 201–209. 10.1016/j.aquatox.2013.01.007 23416413

[jat4370-bib-0017] Kim, J.‐M. , Yang, Y.‐S. , Hong, J. , Chaugule, S. , Chun, H. , van der Meulen, M.C.H , Xu, R. , Greenblatt, M.B. , and Shim, J.H. 2022 “Impaired ERK MAPK activation in mature osteoblasts enhances bone formation via the MTOR pathway.” 2022.01.24.477465.

[jat4370-bib-0018] Knag, A. C. , Verhaegen, S. , Ropstad, E. , Mayer, I. , & Meier, S. (2013). Effects of polar oil related hydrocarbons on steroidogenesis in vitro in H295R cells. Chemosphere, 92(1), 106–115. 10.1016/j.chemosphere.2013.02.046 23561572

[jat4370-bib-0019] Komori, T. (2016). Cell death in chondrocytes, osteoblasts, and osteocytes. International Journal of Molecular Sciences, 17(12). 10.3390/ijms17122045 PMC5187845 27929439

[jat4370-bib-0020] Kuo, T. , Lew, M. J. , Mayba, O. , Harris, C. A. , Speed, T. P. , & Wang, J.‐C. (2012). Genome‐wide analysis of glucocorticoid receptor‐binding sites in myotubes identifies gene networks modulating insulin signaling. Proceedings of the National Academy of Sciences, 109(28), 11160–11165. 10.1073/pnas.1111334109 PMC3396543 22733784

[jat4370-bib-0022] Lee, J.‐M. , Kim, M.‐G. , Byun, J.‐H. , Kim, G.‐C. , Ro, J.‐H. , Hwang, D.‐S. , Choi, B.‐B. , Park, G.‐C. , & Kim, U.‐K. (2017). The effect of biomechanical stimulation on osteoblast differentiation of human jaw periosteum‐derived stem cells. Maxillofacial Plastic and Reconstructive Surgery, 39(1), 7. 10.1186/s40902-017-0104-6 28303237 PMC5337228

[jat4370-bib-0023] Li, C. , Li, F. , Lillico, D. M. E. , Belosevic, M. , Stafford, J. L. , & El‐Din, M. G. (2019). Exposure to organic fraction extracted from oil sands process‐affected water has negligible impact on pregnancy and lactation of mice. Environmental Science & Technology, 53(12), 7083–7094. 10.1021/acs.est.9b01965 31117544

[jat4370-bib-0024] Li, C. , Li, F. , Stafford, J. , Belosevic, M. , & El‐Din, M. G. (2017). The toxicity of oil sands process‐affected water (OSPW): A critical review. Science of the Total Environment, 601–602, 1785–1802. 10.1016/j.scitotenv.2017.06.024 28618666

[jat4370-bib-0025] Li, H. , Li, T. , Fan, J. , Li, T. , Fan, L. , Wang, S. , Weng, X. , Han, Q. , & Zhao, R. C. (2015). MiR‐216a rescues dexamethasone suppression of osteogenesis, promotes osteoblast differentiation and enhances bone formation, by regulating c‐Cbl‐mediated PI3K/AKT pathway. Cell Death & Differentiation, 22(12), 1935–1945. 10.1038/cdd.2015.99 26206089 PMC4816120

[jat4370-bib-0026] Lister, A. , Nero, V. , Farwell, A. , Dixon, D. G. , & Van Der Kraak, G. (2008). Reproductive and stress hormone levels in goldfish (*Carassius auratus*) exposed to oil sands process‐affected water. Aquatic Toxicology (Amsterdam, Netherlands), 87(3), 170–177. 10.1016/j.aquatox.2008.01.017 18336931

[jat4370-bib-0027] Liu, T. M. , & Lee, E. H. (2013). Transcriptional regulatory cascades in Runx2‐dependent bone development. Tissue Engineering. Part B, Reviews, 19(3), 254–263. 10.1089/ten.teb.2012.0527 23150948 PMC3627420

[jat4370-bib-0028] Livak, K. J. , & Schmittgen, T. D. (2001). Analysis of relative gene expression data using real‐time quantitative PCR and the 2(‐delta delta C(T)) method. Methods (San Diego, Calif.), 25(4), 402–408. 10.1006/meth.2001.1262 11846609

[jat4370-bib-0029] Loughery, J. R. , Marentette, J. R. , Frank, R. A. , Mark Hewitt, L. , Parrott, J. L. , & Martyniuk, C. J. (2019). Transcriptome profiling in larval fathead minnow exposed to commercial naphthenic acids and extracts from fresh and aged oil sands process‐affected water. Environmental Science & Technology, 53(17), 10435–10444. 10.1021/acs.est.9b01493 31335129

[jat4370-bib-0030] Mahaffey, A. , & Dubé, M. (2016). Review of the composition and toxicity of oil sands process‐affected water. Environmental Reviews, 25(1), 97–114. 10.1139/er-2015-0060

[jat4370-bib-0031] Marentette, J. R. , Frank, R. A. , Bartlett, A. J. , Gillis, P. L. , Mark Hewitt, L. , Peru, K. M. , Headley, J. V. , Brunswick, P. , Shang, D. , & Parrott, J. L. (2015). Toxicity of naphthenic acid fraction components extracted from fresh and aged oil sands process‐affected waters, and commercial naphthenic acid mixtures, to fathead minnow (*Pimephales promelas*) embryos. Aquatic Toxicology, 164, 108–117. 10.1016/j.aquatox.2015.04.024 25957715

[jat4370-bib-0032] Marentette, J. R. , Sarty, K. , Cowie, A. M. , Frank, R. A. , Mark Hewitt, L. , Parrott, J. L. , & Martyniuk, C. J. (2017). Molecular responses of walleye (*Sander vitreus*) embryos to naphthenic acid fraction components extracted from fresh oil sands process‐affected water. Aquatic Toxicology, 182, 11–19. 10.1016/j.aquatox.2016.11.003 27842271

[jat4370-bib-0033] McClung, M. R. , Grauer, A. , Boonen, S. , Bolognese, M. A. , Brown, J. P. , Diez‐Perez, A. , Langdahl, B. L. , Reginster, J.‐Y. , Zanchetta, J. R. , Wasserman, S. M. , Katz, L. , Maddox, J. , Yang, Y.‐C. , Libanati, C. , & Bone, H. G. (2014). Romosozumab in postmenopausal women with low bone mineral density. The New England Journal of Medicine, 370(5), 412–420. 10.1056/NEJMoa1305224 24382002

[jat4370-bib-0034] Philibert, D. A. , Lyons, D. D. , Qin, R. , Huang, R. , El‐Din, M. G. , & Tierney, K. B. (2019). Persistent and transgenerational effects of raw and ozonated oil sands process‐affected water exposure on a model vertebrate, the zebrafish. Science of the Total Environment, 693, 133611. 10.1016/j.scitotenv.2019.133611 31634996

[jat4370-bib-0035] Plotkin, L. I. , & Bellido, T. (2016). Osteocytic signalling pathways as therapeutic targets for bone fragility. Nature Reviews. Endocrinology, 12(10), 593–605. 10.1038/nrendo.2016.71 PMC6124897 27230951

[jat4370-bib-0036] Poetsch, F. , Henze, L. A. , Estepa, M. , Moser, B. , Pieske, B. , Lang, F. , Eckardt, K.‐U. , Alesutan, I. , & Voelkl, J. (2020). Role of SGK1 in the osteogenic transdifferentiation and calcification of vascular smooth muscle cells promoted by hyperglycemic conditions. International Journal of Molecular Sciences, 21(19), 7207. 10.3390/ijms21197207 33003561 PMC7583813

[jat4370-bib-0037] Rauch, A. , Seitz, S. , Baschant, U. , Schilling, A. F. , Illing, A. , Stride, B. , Kirilov, M. , Mandic, V. , Takacz, A. , Schmidt‐Ullrich, R. , Ostermay, S. , Schinke, T. , Spanbroek, R. , Zaiss, M. M. , Angel, P. E. , Lerner, U. H. , David, J.‐P. , Reichardt, H. M. , Amling, M. , … Tuckermann, J. P. (2010). Glucocorticoids suppress bone formation by attenuating osteoblast differentiation via the monomeric glucocorticoid receptor. Cell Metabolism, 11(6), 517–531. 10.1016/j.cmet.2010.05.005 20519123

[jat4370-bib-0038] Rodríguez‐Estival, J. , & Smits, J. E. G. (2016). Small mammals as sentinels of oil sands related contaminants and health effects in northeastern Alberta, Canada. Ecotoxicology and Environmental Safety, 124, 285–295. 10.1016/j.ecoenv.2015.11.001 26555251

[jat4370-bib-0039] Rogers, V. V. , Liber, K. , & MacKinnon, M. D. (2002). Isolation and characterization of naphthenic acids from Athabasca oil sands tailings pond water. Chemosphere, 48(5), 519–527. 10.1016/S0045-6535(02)00133-9 12146630

[jat4370-bib-0040] Seeman, E. (2003). Periosteal bone formation—A neglected determinant of bone strength. The New England Journal of Medicine, 349(4), 320–323. 10.1056/NEJMp038101 12878736

[jat4370-bib-0041] Silva, B. C. , & Bilezikian, J. P. (2015). Parathyroid hormone: Anabolic and catabolic actions on the skeleton. Current Opinion in Pharmacology, 22, 41–50. 10.1016/j.coph.2015.03.005 25854704 PMC5407089

[jat4370-bib-0042] Sonne, C. , Dyck, M. , Rigét, F. F. , Jensen, J.‐E. B. , Hyldstrup, L. , Letcher, R. J. , Kim Gustavson, M. , Gilbert, T. P. , & Dietz, R. (2015). Penile density and globally used chemicals in Canadian and Greenland polar bears. Environmental Research, 137, 287–291. 10.1016/j.envres.2014.12.026 25601730

[jat4370-bib-0043] Stein, E. M. , Silva, B. C. , Boutroy, S. , Zhou, B. , Wang, J. , Udesky, J. , Zhang, C. , McMahon, D. J. , Romano, M. , Dworakowski, E. , Costa, A. G. , Cusano, N. , Irani, D. , Cremers, S. , Elizabeth Shane, X. , Guo, E. , & Bilezikian, J. P. (2013). Primary hyperparathyroidism is associated with abnormal cortical and trabecular microstructure and reduced bone stiffness in postmenopausal women. Journal of Bone and Mineral Research: The Official Journal of the American Society for Bone and Mineral Research, 28(5), 1029–1040. 10.1002/jbmr.1841 23225022 PMC3631282

[jat4370-bib-0044] Stockley, P. , Ramm, S. A. , Sherborne, A. L. , Thom, M. D. F. , Paterson, S. , & Hurst, J. L. (2013). Baculum morphology predicts reproductive success of male house mice under sexual selection. BMC Biology, 11, 66. 10.1186/1741-7007-11-66 23800051 PMC3693876

[jat4370-bib-0045] Thomas, P. J. , Newell, E. E. , Eccles, K. , Holloway, A. C. , Idowu, I. , Xia, Z. , Hassan, E. , Tomy, G. , & Quenneville, C. (2021). Co‐exposures to trace elements and polycyclic aromatic compounds (PACs) impacts North American River otter (*Lontra canadensis*) baculum. Chemosphere, 265, 128920. 10.1016/j.chemosphere.2020.128920 33213878

[jat4370-bib-0046] Vander Meulen, I. J. , Schock, D. M. , Parrott, J. L. , Mundy, L. J. , Pauli, B. D. , Peru, K. M. , McMartin, D. W. , & Headley, J. V. (2021). Characterization of naphthenic acid fraction compounds in water from Athabasca oil sands wetlands by orbitrap high‐resolution mass spectrometry. Science of the Total Environment, 780, 146342. 10.1016/j.scitotenv.2021.146342 33770601

[jat4370-bib-0047] Wang, D. , Christensen, K. , Chawla, K. , Xiao, G. , Krebsbach, P. H. , & Franceschi, R. T. (1999). Isolation and characterization of MC3T3‐E1 preosteoblast subclones with distinct in vitro and in vivo differentiation/mineralization potential. Journal of Bone and Mineral Research, 14(6), 893–903. 10.1359/jbmr.1999.14.6.893 10352097

[jat4370-bib-0048] Weinstein, R. S. , Jilka, R. L. , Parfitt, A. M. , & Manolagas, S. C. (1998). Inhibition of osteoblastogenesis and promotion of apoptosis of osteoblasts and osteocytes by glucocorticoids. Potential mechanisms of their deleterious effects on bone. The Journal of Clinical Investigation, 102(2), 274–282. 10.1172/JCI2799 9664068 PMC508885

[jat4370-bib-0049] Wu, C. , De Visscher, A. , & Gates, I. D. (2019). On naphthenic acids removal from crude oil and oil sands process‐affected water. Fuel, 253, 1229–1246. 10.1016/j.fuel.2019.05.091

[jat4370-bib-0050] Xu, S. , Guo, R. , Li, P.‐Z. , Ke Li, Y. , Yan, J. C. , Wang, G. , Brand‐Saberi, B. , Yang, X. , & Cheng, X. (2019). Dexamethasone interferes with osteoblasts formation during osteogenesis through altering IGF‐1‐mediated angiogenesis. Journal of Cellular Physiology, 234(9), 15167–15181. 10.1002/jcp.28157 30671960

